# Supplementary material for: Patterns of Glycemic Variability During a Diabetes Self-Management Educational Program
Source: Med Sci (Basel). 2019 Mar 25;7(3):52. doi: 10.3390/medsci7030052 (PMC6473237; doi:10.3390/medsci7030052)
Supplement: Supplementary file 1 [file medsci-07-00052-s001.pdf]

Additional Tables

Table S1: Day-wise number of observations and mean glucose values across the study groups

	Acceptable Control		Not Controlled		Optimal Control	
	No of Observations (CGMS Readings)	Glucose (mg/dL)	No of Observations (CGMS Readings)	Glucose (mg/dL)	No of Observations (CGMS Readings)	Glucose (mg/dL)
Day 2 (Baseline)	960	132.224	1920	211.1557	1056	119.3286
Day 3	960	122.2219	1920	196.1875	1056	112.4801
Day 4	960	117.8521	1907	189.6371	1056	115.3172
Day 5	960	111.6219	1920	187.7849	1056	112.5047
Day 6	960	109.1719	1920	182.1031	1056	106.7169
Day 7 (Midpoint)	960	104.6698	1915	179.6757	1056	107.4025
Day 8	960	103.9094	1920	173.9479	1056	108.5076
Day 9	960	107.0938	1920	167.7115	1056	111.1676
Day 10	960	108.2354	1917	161.3693	1056	112.2358
Day 11	864	108.1424	1920	158.1688	960	100.9698
Day 12	864	104.7419	1824	149.9803	960	98.94583
Day 13 (End)	864	99.67708	1824	139.2582	864	97.39468

Table S2: The number of observations of glucose values of study participants before and after removing the observations from the first and last day of the study.

S.No	ID	No of Observations		Inclusion	Remark
		Before	After		
1	10013	1231	1152	Included	
2	10020	1237	1152	Included	
3	10032	1056		Excluded	Clustering of Missing Values in continuity leading to misappropriation by imputation
4	10033	675		Excluded	Insufficient Observations (8 days)
5	10036	1241	1152	Included	
6	10038	1240	1152	Included	
7	10039	1239	1152	Included	
8	10040	1232	1152	Included	
9	10041	1241	1152	Included	
10	10042	1240	1152	Included	
11	10043	1240	1152	Included	
12	10044	1240	1152	Included	
13	10045	1233	1152	Included	
14	10046	1232	1152	Included	
15	10048	1241	1152	Included	
16	10049	1240	1152	Included	
17	10050	1166	1056	Included	Complete spectrum of CGMS readings without any missing values for 11 days inclusive of Baseline (Day 1) and Mid-Intervention (Day 7)
18	10052	1239	1152	Included	
19	10053	1239	1152	Included	
20	10054	1240	1152	Included	
21	10056	1234	1152	Included	
22	10057	1234	1152	Included	
23	10058	1238	1152	Included	
24	10064	1237	1152	Included	
25	10068	1238	1152	Included	
26	10076	1237	1152	Included	
27	10082	1236	1152	Included	
28	10090	952		Excluded	283 Missing Values in continuity leading to misappropriation by imputation
29	10104	1238	1152	Included	
30	10118	979	864	Included	Complete spectrum of CGMS readings without any missing values for 9 days inclusive of Baseline (Day 1) and Mid-Intervention (Day 7)
31	10123	1239	1152	Included	
32	10124	1239	1152	Included	
33	10128	1235	1152	Included	
34	10139	1237	1152	Included	
35	10140	1236	1152	Included	
36	10141	997	864	Included	Complete spectrum of CGMS readings without any missing values for 9 days inclusive of Baseline (Day 1) and Mid-Intervention (Day 7)
37	10142	1184	1152	Included	
38	10143	1235		Excluded	CGMS Sensor (Mechanical Error - Implausible Glucose Values)
39	10144	587		Excluded	Insufficient Observations (8 days)
40	10145	1235	1152	Included	
41	10146	1237	1152	Included	
42	10147	1238	1152	Included	
43	10148	1238	1152	Included	
44	10149	1236	1152	Included	
45	10150	1141	1056	Included	Complete spectrum of CGMS readings without any missing values for 11 days inclusive of Baseline (Day 1) and Mid-Intervention (Day 7)
46	10152	1237	1152	Included	

The table above shows the overview of the 46 initial study participants and their contribution to the data. The participants with >20% missing values have been excluded. Data of those study participants having <20% missing values were meticulously analysed and imputed accordingly.

Table S3. Trend of mean glucose values with reference to time

	Optimal control (n=11)	*Acceptable control (n=10)*	*Poor control (n=20)*
Mean(SD) HbA1c	6.55 (0.32)	7.50 (0.25)	9.51 (1.22)
Baseline			
Mean glucose(SD)	115.90 (34.15)	127.22 (50.02)	203.67 (77.43)
Coefficient of variation	29.46	39.32	38.02
Mid			
Mean glucose(SD)	107.96 (34.15)	104.29 (27.62)	176.81 (62.22)
Coefficient of variation	31.64	26.48	35.19
End			
Mean glucose(SD)	98.21 (34.86)	102.21 (25.50)	144.62 (53.26)
Coefficient of variation	35.49	24.94	36.83
r-ANOVA			
F-statistic	6.795	15.779	11.948
p-value#	0.0066	<0.0001	0.0005

# p-value computed for mean glucose readings

Data Cleaning and Manipulation

The following code describes in detail the data cleaning process.

Loading of required packages

```
# Load necessary packages
require(readxl)
```

```
## Loading required package: readxl
```

```
require(dplyr)
```

```
## Loading required package: dplyr

##
## Attaching package: 'dplyr'

## The following objects are masked from 'package:stats':
##
##     filter, lag

## The following objects are masked from 'package:base':
##
##     intersect, setdiff, setequal, union
```

```
require(xts)
```

```
## Loading required package: xts

## Loading required package: zoo

##
## Attaching package: 'zoo'

## The following objects are masked from 'package:base':
##
##     as.Date, as.Date.numeric

##
## Attaching package: 'xts'

## The following objects are masked from 'package:dplyr':
##
##     first, last
```

```
require(lubridate)
```

```
## Loading required package: lubridate

##
## Attaching package: 'lubridate'

## The following object is masked from 'package:base':
##
##     date
```

```
require(data.table)
```

```
## Loading required package: data.table
```

```
##
## Attaching package: 'data.table'

## The following objects are masked from 'package:lubridate':
##
##     hour, isoweek, mday, minute, month, quarter, second, wday,
##     week, yday, year

## The following objects are masked from 'package:xts':
##
##     first, last

## The following objects are masked from 'package:dplyr':
##
##     between, first, last
```

```r
require(imputeTS)
```

```
## Loading required package: imputeTS

##
## Attaching package: 'imputeTS'

## The following object is masked from 'package:zoo':
##
##     na.locf
```

```r
require(forecast)
```

```
## Loading required package: forecast
```

```r
require(astsa)
```

```
## Loading required package: astsa

##
## Attaching package: 'astsa'

## The following object is masked from 'package:forecast':
##
##     gas
```

```r
require(magicfor)
```

```
## Loading required package: magicfor
```

```r
require(ggplot2)
```

```
## Loading required package: ggplot2
```

```r
require(kableExtra)
```

```
## Loading required package: kableExtra
```

Reading in the excel file

The path of the excel file is saved as an object `file_path`. The file path is then passed through the `read_excel` function and the resulting dataset object is saved as `df`.

Variable manipulation

The following code manipulates the `df$Time` variable as a character and then coherces it into a `POSIXct` object with a `%Y-%m-%d %H:%M:%S` format. The variable `df$DC_Number` is coherced into a factor variable and saved as `df$dc_number` for ease of use. Similar renaming was done for `df$Glucose`.

```
# Create workable Time and Date variables
df$Time <- as.character.Date(df$Time)
df$Time <- as.POSIXct(df$Time,format="%Y-%m-%d %H:%M:%S")
df$dc_number <- factor(df$DC_Number)
df$glucose <- df$Glucose
```

Dataframe manipulation

In order to ensure uniformity, the readings from the frist day and the last day of each individual are discarded. The following code describes the method how it is acheived:

Spliting of the dataframe

The dataframe `df` is split by the factor variable `df$dc_number`, resulting in a list of 46 dataframes which is saved in an object `data_list` of `list` class.

```
# Split dataframe by individual
data_list <- split(df,df$dc_number)
```

A vector named `date_index` has been created by cohercing the `Date` variable into a factor and then saving it as an integer. This gives the vector of `date_index` which contains values from 1 – 14.

Next, a logical vector `date_index_logical` was created which was `FALSE` for the minimum and maximum value of the `date_index` variable. This was saved as a list named `data.1`.

```
# Create date_index object
data <- lapply(data_list, transform, date_index = as.integer(factor(Date)))
data.1 <- lapply(data, transform, date_index_logical = ifelse((date_index==min(date_index)|(date_index=
```

Merging the dataframe

The dataframes that have been split are then merged again into a single data frame with the name `df` by using the `do.call` function and `rbind`. A backup of the data frame is also created and named as `df.backup`.

```
df <- do.call("rbind",data.1)
df.backup.1 <- df # Make backup
```

Discarding the first and last day observations

```
df <- df[which(df$date_index_logical=="TRUE"),]
df$date_index <-  df$date_index - 1
```

Now, `df` contains the observations of all 46 individuals but the first and last day observations removed. The number of observations in the dataset by date are tabulated in the table below:

```
# Tabulate number of obs per day per individual
d<- df %>%
  group_by(dc_number,date_index) %>%
  summarise(number = n())
```

Visualising individuals according to the number of days

```
# number of total days
magic_for(print, silent = TRUE)
x <- as.numeric(levels(df$dc_number))
y <- NULL
for(i in unique(x)){
  y[i] <- length(levels(factor(df[which(df$dc_number==i),]$Date)))
  print(y[i])
}
d1 <- magic_result_as_dataframe()
d1 <- cbind.data.frame(ID = levels(df$dc_number), days = d1[,2])
d1$expected <- d1$days*96

# Plot number of days by individual
ggplot(d1, aes(x=reorder(ID,-days),y = days, fill = factor(days))) +
  geom_bar(stat = "identity") +
  coord_flip() +
  scale_fill_brewer(palette="Pastel2") +
  scale_y_continuous()
```

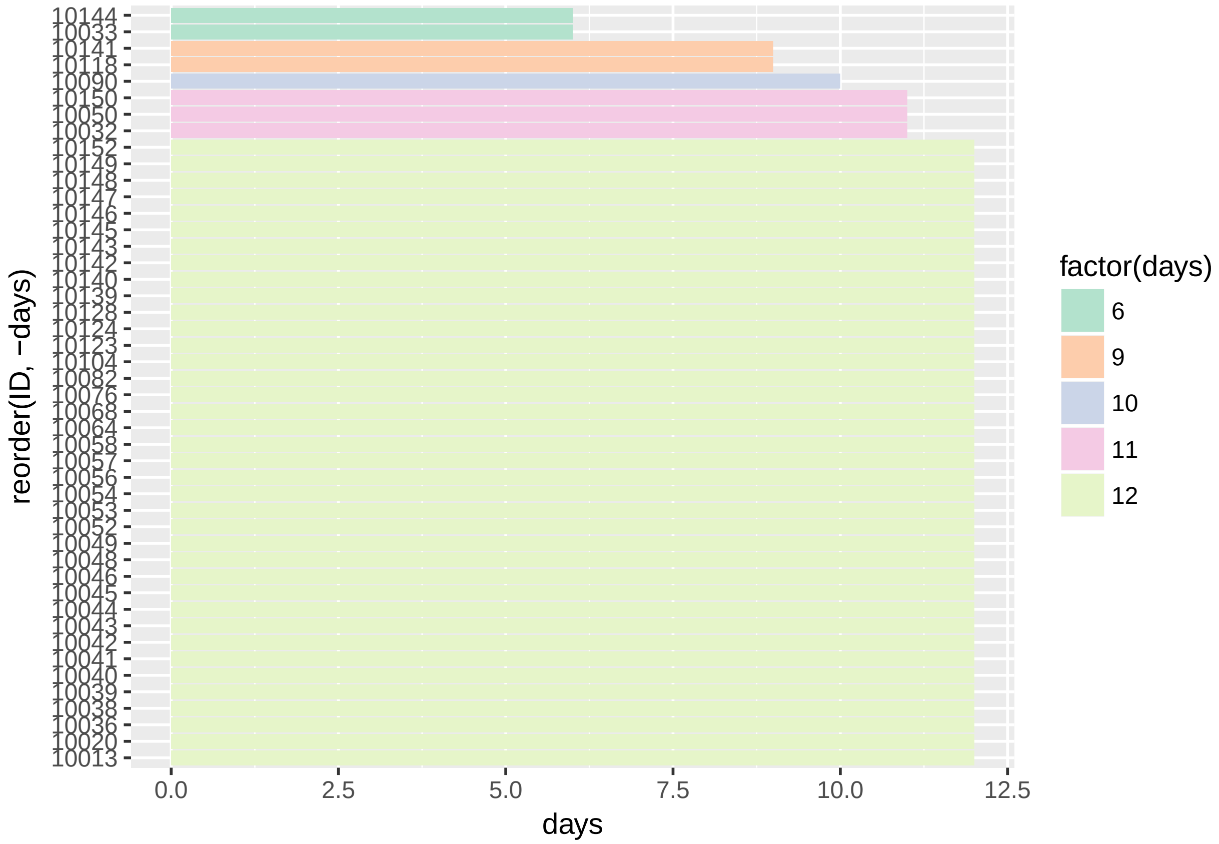

The above graph shows the individuals according to the number total days of readings. It was found that 2 individuals i.e. 10144 & 10033 who were removed from the further analysis

```
df.backup.2 <- df
df <- df[which(df$dc_number!="10144"),]
df <- df[which(df$dc_number!="10033"),]
```

```
df <- droplevels.data.frame(df)
```

The following plot shows the percent of missing values on y axis and index on the x axis. The missing data exceeds the 20% in the following individuals: 10032, 10090 and 10142.

```
d.1 <- df %>%
  group_by(dc_number, date_index) %>%
  summarize(number = n())

d.1$missing_percent <- (1 - (d.1$number/96))*100

plot(d.1$missing_percent)
abline(h=20, col=2)
with(d.1, text(missing_percent, labels = paste(d.1$dc_number,d.1$date_index), pos = 4))
```

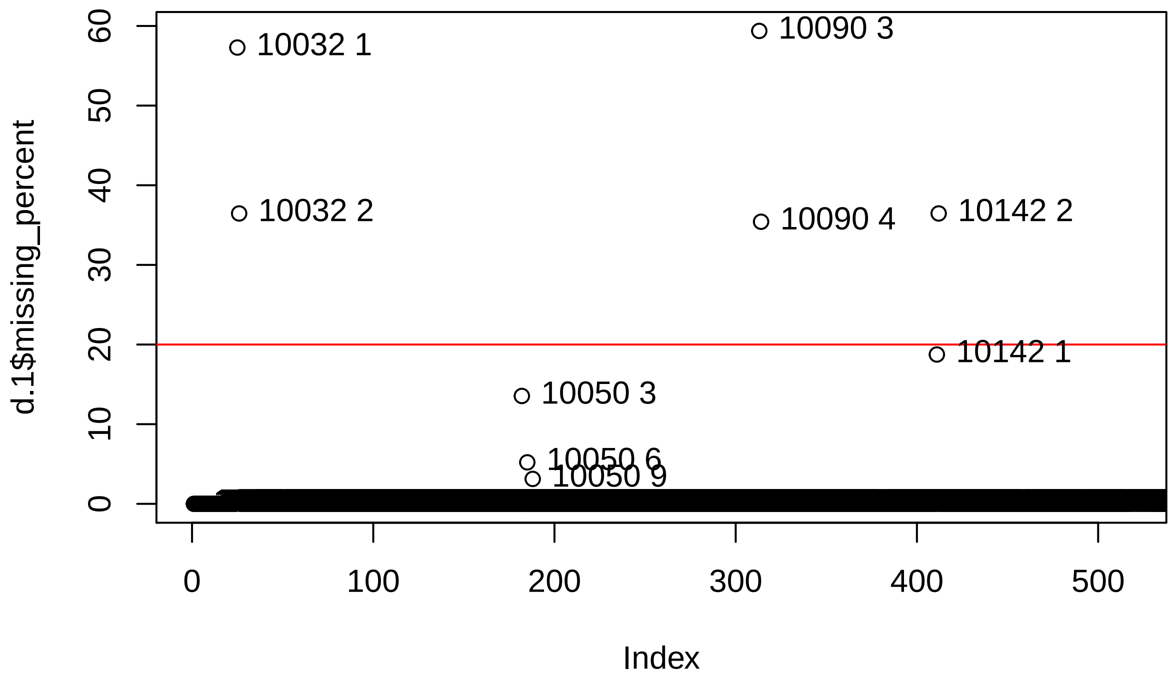

So these individuals are removed from any further analysis.

```
df.backup.3 <- df
df <- df[which(df$dc_number!="10032"),]
df <- df[which(df$dc_number!="10090"),]
df <- df[which(df$dc_number!="10142"),]
df <- droplevels.data.frame(df)
```

One individuals i.e. `dc_number` 10050 have misssing <20% of the total 96 daily observations. The details of this is shown in the table below.

```
d.x <- df %>%
        group_by(dc_number, Date) %>%
```

```r
        summarize(number = n())
table(d.x$number)
```

```
##
##  83  91  93  96
##   1   1   1 481
```

```r
d.x1 <- d.x[which(d.x$number!=96),]
```

Identify the missing indices and impute the values

```r
# Split the dataset as individuals to be imputed and those that need not be imputed
#df[which((df$dc_number=="10050") & (df$date_index==3)),] #10050 2017-04-18
#df[which((df$dc_number=="10050") & (df$date_index==6)),] #10050 2017-04-21
#df[which((df$dc_number=="10050") & (df$date_index==9)),] #10050 2017-04-24
```

Remove the individual 10050 from dataset for imputation

```r
df.to.impute <- df[which(df$dc_number=="10050"),]
df.other <- df[which(df$dc_number!="10050"),]
```

Visualise the missing values in the dataset

```r
# Manipulation of the df.to.impute
df.to.impute1 <- df.to.impute[which(df.to.impute$date_index==3),]
df.to.impute2 <- df.to.impute[which(df.to.impute$date_index==6),]
df.to.impute3 <- df.to.impute[which(df.to.impute$date_index==9),]


#imputation required for date indices are 3, 6, 9
# The working code for this is still in the process..
# impute_list[[3]]$Time
# impute_list[[6]]$Time
# impute_list[[9]]$Time

start_time1 <- min(df.to.impute1$Time)
end_time1 <- max(df.to.impute1$Time)
output1 <- seq.POSIXt(start_time1, end_time1, by = "15 min")
y <- as.data.frame(output1)

# Indices of the missing values
which(is.na(match(y$output1,df.to.impute1$Time)))
```

```
##  [1] 55 56 57 58 59 62 63 64 65 66 67 68 69
```

```r
y$glucose <- df.to.impute1$glucose[match(y$output1,df.to.impute1$Time)]
y <- as.data.table(y)
y <- as.xts(y)

# imputation with k = 10
y1 <- na.ma(y, k = 10, weighting = "exponential")
```

```r
# Details about the missing values
statsNA(y)
```

```
## [1] "Length of time series:"
## [1] 96
## [1] "-------------------------"
## [1] "Number of Missing Values:"
## [1] 13
## [1] "-------------------------"
## [1] "Percentage of Missing Values:"
## [1] "13.5%"
## [1] "-------------------------"
## [1] "Stats for Bins"
## [1] "  Bin 1 (24 values from 1 to 24) :      0 NAs (0%)"
## [1] "  Bin 2 (24 values from 25 to 48) :      0 NAs (0%)"
## [1] "  Bin 3 (24 values from 49 to 72) :      13 NAs (54.2%)"
## [1] "  Bin 4 (24 values from 73 to 96) :      0 NAs (0%)"
## [1] "-------------------------"
## [1] "Longest NA gap (series of consecutive NAs)"
## [1] "8 in a row"
## [1] "-------------------------"
## [1] "Most frequent gap size (series of consecutive NA series)"
## [1] "8 NA in a row (occuring 1 times)"
## [1] "-------------------------"
## [1] "Gap size accounting for most NAs"
## [1] "8 NA in a row (occuring 1 times, making up for overall 8 NAs)"
## [1] "-------------------------"
## [1] "Overview NA series"
## [1] "  5 NA in a row: 1 times"
## [1] "  8 NA in a row: 1 times"
```

```r
#4 figures arranged in 2 row and 1 columns
par(mfrow=c(2,1))
# Plot the missing values
plotNA.distribution(ts(y), main = "With NAs, before imputation")
# Plot after imputation done
plotNA.distribution(ts(y1), main = "Without NAs, after imputation")
```

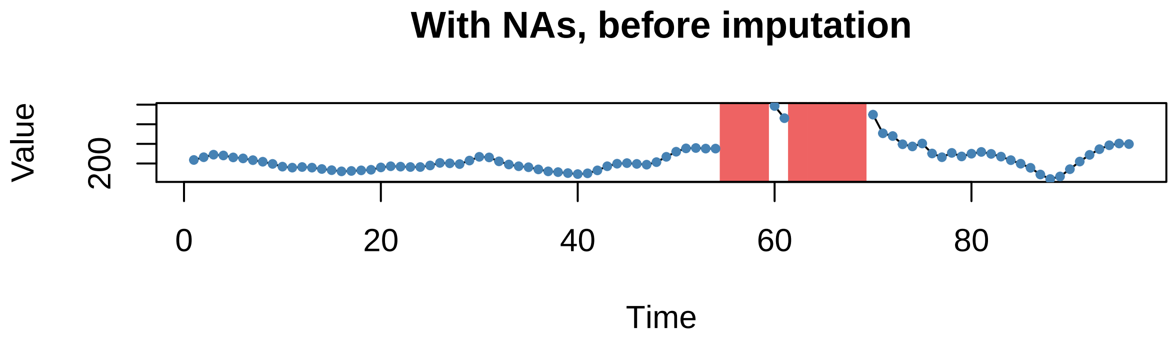

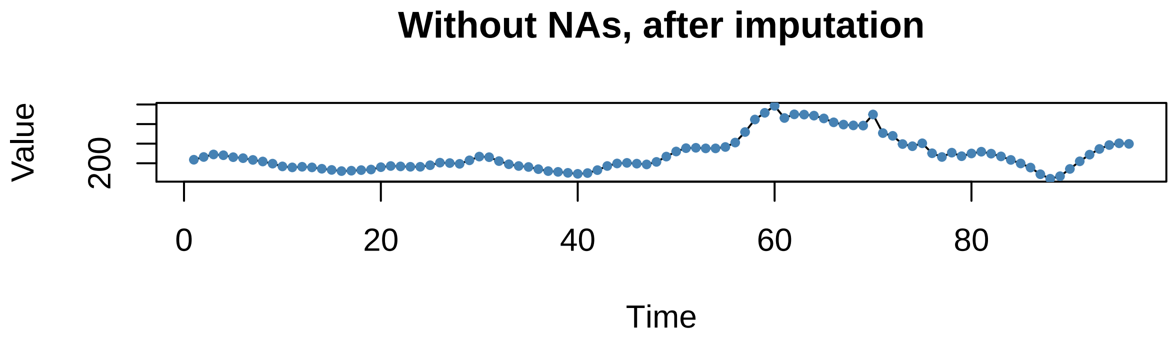


```r
# Imputed dataframe
# cbind.data.frame(y,y1)
```

```
## [1] 22 23 24 25 50
```

```
## [1] "Length of time series:"
## [1] 96
## [1] "-------------------------"
## [1] "Number of Missing Values:"
## [1] 5
## [1] "-------------------------"
## [1] "Percentage of Missing Values:"
## [1] "5.21%"
## [1] "-------------------------"
## [1] "Stats for Bins"
## [1] "  Bin 1 (24 values from 1 to 24) :      3 NAs (12.5%)"
## [1] "  Bin 2 (24 values from 25 to 48) :      1 NAs (4.17%)"
## [1] "  Bin 3 (24 values from 49 to 72) :      1 NAs (4.17%)"
## [1] "  Bin 4 (24 values from 73 to 96) :      0 NAs (0%)"
## [1] "-------------------------"
## [1] "Longest NA gap (series of consecutive NAs)"
## [1] "4 in a row"
## [1] "-------------------------"
## [1] "Most frequent gap size (series of consecutive NA series)"
## [1] "4 NA in a row (occuring 1 times)"
## [1] "-------------------------"
## [1] "Gap size accounting for most NAs"
```

```
## [1] "4 NA in a row (occuring 1 times, making up for overall 4 NAs)"
## [1] "-------------------------"
## [1] "Overview NA series"
## [1] "  1 NA in a row: 1 times"
## [1] "  4 NA in a row: 1 times"
```

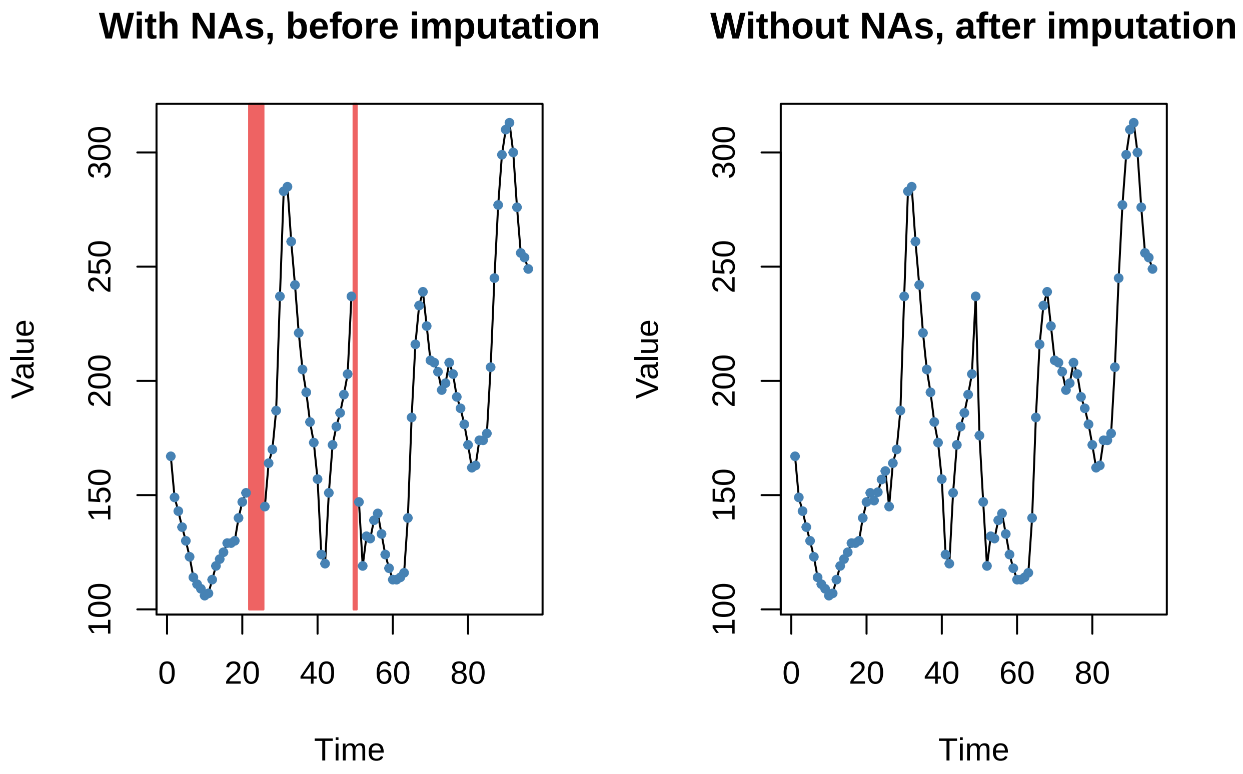


```r
# Imputed dataframe
# cbind.data.frame(y,y2)
```

```
## [1] 31 32 33
```

```
## [1] "Length of time series:"
## [1] 96
## [1] "-------------------------"
## [1] "Number of Missing Values:"
## [1] 3
## [1] "-------------------------"
## [1] "Percentage of Missing Values:"
## [1] "3.12%"
## [1] "-------------------------"
## [1] "Stats for Bins"
## [1] "  Bin 1 (24 values from 1 to 24) :      0 NAs (0%)"
## [1] "  Bin 2 (24 values from 25 to 48) :      3 NAs (12.5%)"
## [1] "  Bin 3 (24 values from 49 to 72) :      0 NAs (0%)"
## [1] "  Bin 4 (24 values from 73 to 96) :      0 NAs (0%)"
## [1] "-------------------------"
## [1] "Longest NA gap (series of consecutive NAs)"
```

```
## [1] "3 in a row"
## [1] "-------------------------"
## [1] "Most frequent gap size (series of consecutive NA series)"
## [1] "3 NA in a row (occuring 1 times)"
## [1] "-------------------------"
## [1] "Gap size accounting for most NAs"
## [1] "3 NA in a row (occuring 1 times, making up for overall 3 NAs)"
## [1] "-------------------------"
## [1] "Overview NA series"
## [1] "  3 NA in a row: 1 times"
```

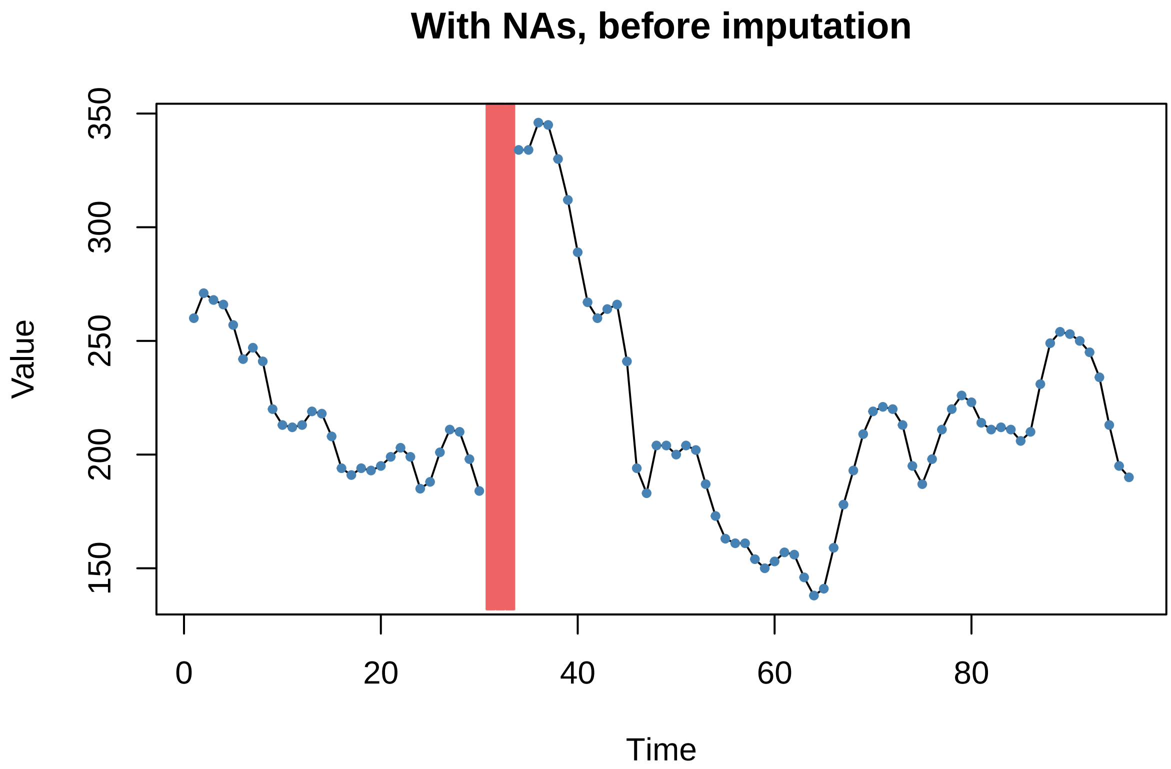

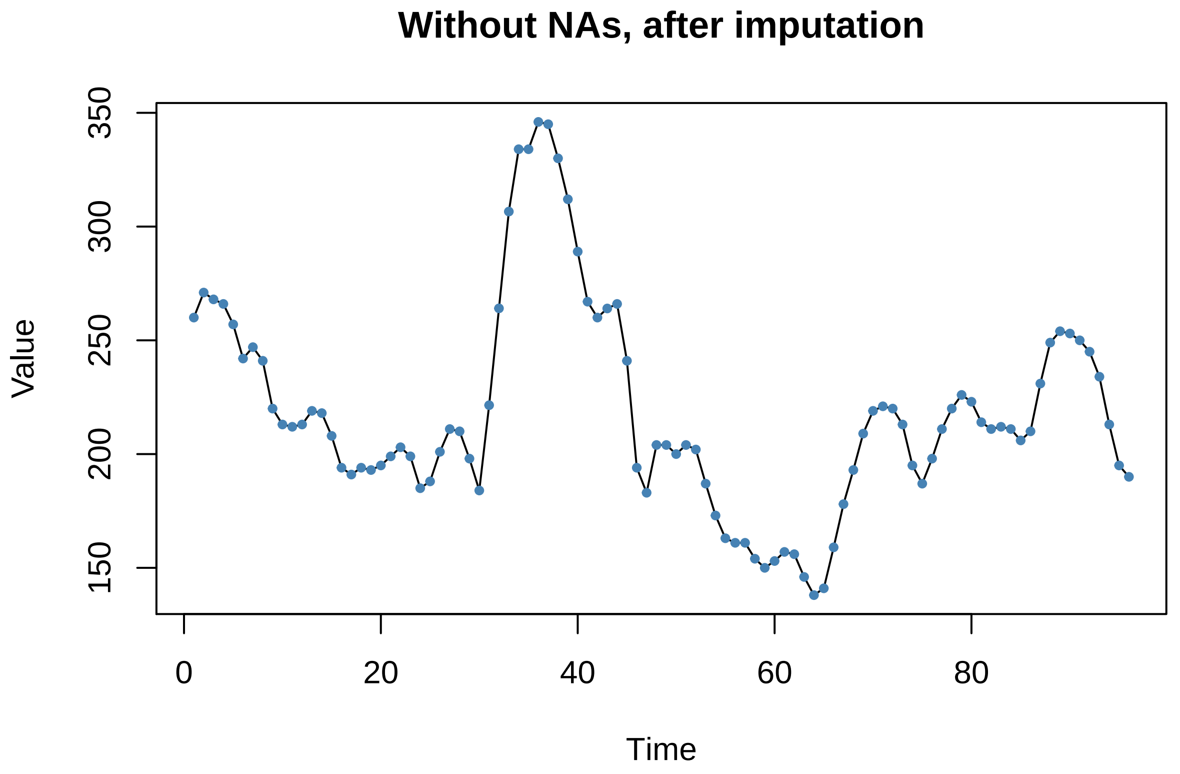


```
# Imputed dataframe
# cbind.data.frame(y,y3)
```

R Code

This document presents the R codes and data for reproducing our analysis result as described in the paper. To start, first download the CGM data and R code for subsequent analysis and plotting into a folder. The data files are available as part of the paper supplementary appendix.

Contents

1 Load Packages **1**

2 Load Dataset and Create workable Time and Date variables **1**

3 Functions for measures of glycemic variability (GV) **2**
- 3.1 MODD . . . 2
- 3.2 MAGE . . . 2
- 3.3 CONGA . . . 3
- 3.4 HBGI . . . 3
- 3.5 LBGI . . . 3

4 Estimation of the Glycemic Variability Parameters **3**

5 Plots **4**
- 5.1 Composite Graph . . . 4
- 5.2 Pioncare Plot . . . 6

---

1 Load Packages

```
library(dplyr)
library(ggplot2)
library(readxl)
library(xts)
library(lubridate)
library(readr)
library(raster)
library(stringr)
library(reshape2)
library(ggthemes)
library(pander)
library(forecast)
library(gridExtra)
```

2 Load Dataset and Create workable Time and Date variables

```
df <- read_csv("C:/Users/Arun/Dropbox/CGM_Study/data_analysis/Final_Data_with_Imputations.csv")
# The path to the data should be changed depending on its location on the
```

```r
# computer

# Data Manipulation
df$Time <- as.character.Date(df$Time)
df$Time <- as.POSIXct(df$Time, format = "%Y-%m-%d %H:%M:%S")
df$date <- as.Date(df$Time, format = "%Y-%m-%d %H:%M")
df$dc_number <- factor(df$dc_number)
df$glucose <- df$Glucose

# Create backup
df_backup <- df

# Split dataframe
data_list <- split.data.frame(df, df$dc_number)
data <- lapply(data_list, transform, id = index(Time))
data <- lapply(data, transform, hrly = id%%4 == 0)
data <- lapply(data, subset, hrly == TRUE)

# Merge dataframes
df <- do.call("rbind", data)
```

3 Functions for measures of glycemic variability (GV)

The R code for estimation of the measures of glycemic variablity: - mean of daily differences (MODD) - mean amplitude of glycemic excursions (MAGE) - continuous overall net glycemic action (CONGA) - high blood glucose index (HBGI) - low blood glucose index (LBGI)

3.1 MODD

```r
# MODD
modd <- function(x){
  x.1 <- diff(x,lag = 96,differences = 1)
  x.2 <- abs(x.1)
  x.3 <- mean(x.2)
  return(x.3)
}
```

3.2 MAGE

```r
# MAGE
mage <-   function(x){
  d.glu <- abs(diff(x))
  sd.glu <- sd(d.glu)
  d1 <- d.glu >= sd.glu
  mage<- mean(x[which(d1 == T)])
  print(mage)
}
```

3.3 CONGA

```
# CONGA
conga <-   function(x){
  d.glu <- abs(diff(x))
  conga <- sd(d.glu)
  print(conga)
}
```

3.4 HBGI

```
# HBGI
hbgi <-   function(x){
  f_bg <- 1.509*(((log(x))^1.084)-5.381)
  r_bg <- 10*(f_bg^2)
  s_bg <- f_bg>=0
  rh_bg <- r_bg[which(s_bg==T)]
  hbgi <- mean(rh_bg)
  print(hbgi)
}
```

3.5 LBGI

```
# LBGI
lbgi <-   function(x){
  f_bg <- 1.509*(((log(x))^1.084)-5.381)
  r_bg <- 10*(f_bg^2)
  s_bg <- f_bg<=0
  rl_bg <- r_bg[which(s_bg==T)]
  lbgi <- mean(rl_bg)
  print(lbgi)
}
```

4 Estimation of the Glycemic Variability Parameters

The following R-code estimates the measures of glycemic variablility and saves them in a separate *.csv file in the folder.

```
# Calculate MAGE & CONGA by individual and date
d <- df %>% group_by(dc_number, date_index) %>% summarise(MAGE = mage(glucose),
    CONGA = conga(glucose))
write.csv(d, "MAGE_CONGA.csv")

# Recall original dataset
df <- df_backup

# Calculate LBGI & HBGI by individual
d <- df %>% group_by(dc_number) %>% summarise(LGBI = lbgi(glucose), HGBI = hbgi(glucose))
write.csv(d, "LBGI_HBGI.csv")
```

```r
# Calculate MODD
d <- df %>% group_by(dc_number, Baseline_Group) %>% filter(n() > 1) %>% summarize(MODD = modd(glucose))
write.csv(d, "MODD.csv")

# Calculate Coefficient of Variation
d <- df %>% group_by(date_index, Baseline_Group) %>% filter(n() > 1) %>% summarize(n = n(),
    CV = cv(glucose))
write.csv(d, "CV.csv")
```

5 Plots

5.1 Composite Graph

The composite graph of the measures of glycemic variability was plotted using the `facet_wrap` argument of the `ggplot2` package. This was plotted separately for coeffecient of variation (CV), mean of daily differences (MODD), mean amplitude of glycemic excursions (MAGE), continuous overall net glycemic action (CONGA) and the high blood glucose index (HBGI) & low blood glucose index (LBGI).

5.1.1 CV, MAGE, MODD & CONGA

```r
# Load Dataset
df <- read_excel("C:/Users/Arun/Dropbox/CGM_Study/data_analysis/composite_figure/baseline_data_new_with
# The path to the data should be changed depending on its location on the computer

# Calculate percentage change
df$GV_Index <- factor(df$GV_Index, levels = c("CV","MODD", "MAGE", "CONGA"))
df$Time <- factor(df$Time, levels = c("Baseline","Mid","End"))
df$Group <- factor(df$Group, levels = c("Optimal", "Acceptable","Poor"))
df$pct_change <- df$pct_change - 100
df$`Percentage Change` <- df$pct_change

# Plot
ggplot(data=df, aes(x=Time, y=`Percentage Change`, group=Group, colour = Group)) +
  geom_line(aes(linetype=Group), size = 1) +
  geom_point() +
  scale_color_manual(values=c("green4", "orange1", "red3")) +
  facet_wrap( .~ GV_Index,dir = "v", scales = "fixed")
```

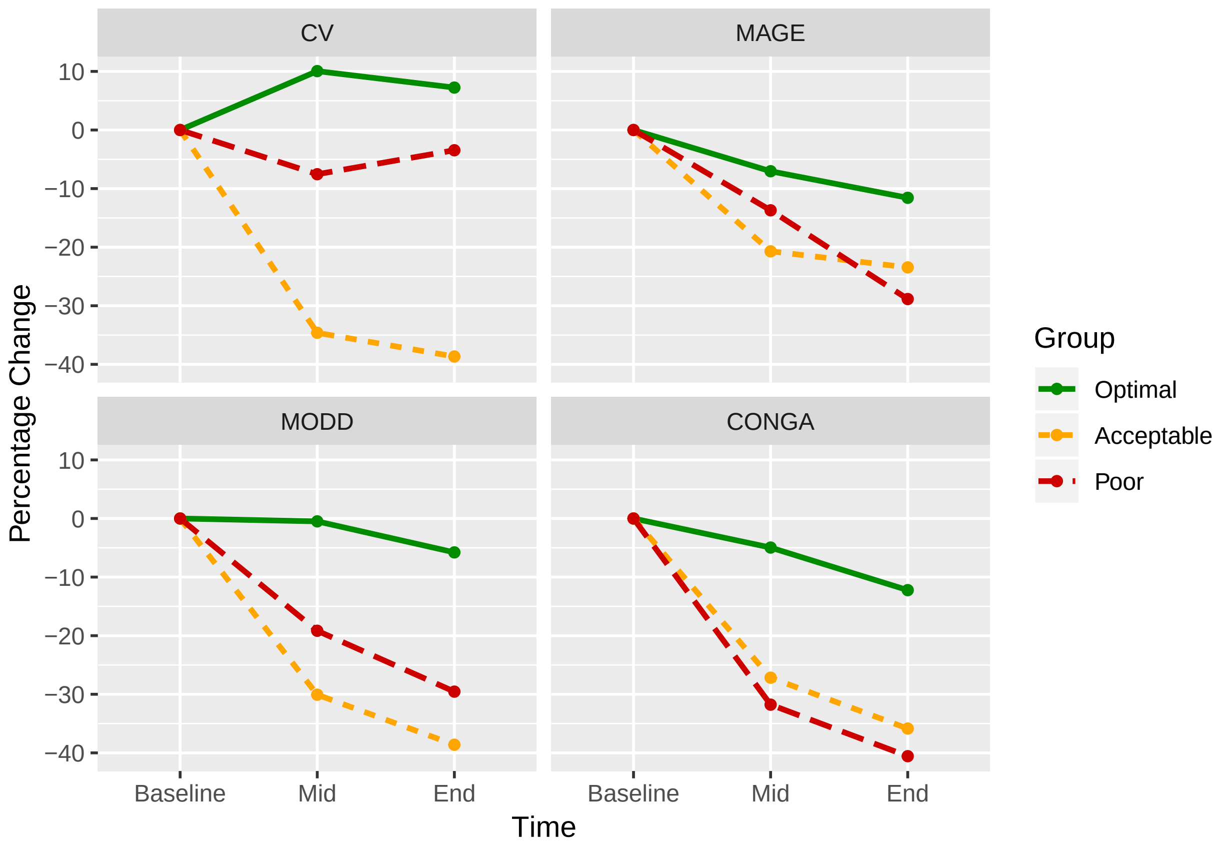

5.1.2 HBGI & LBGI

```
# Load Dataset
df <- read_excel("C:/Users/Arun/Dropbox/CGM_Study/data_analysis/composite_figure/baseline_data_new_only_
# The path to the data should be changed depending on its location on the
# computer

# Calculate percentage change
df$GV_Index <- factor(df$GV_Index, levels = c("HBGI", "LBGI"))
df$Time <- factor(df$Time, levels = c("Baseline", "Mid", "End"))
df$Group <- factor(df$Group, levels = c("Optimal", "Acceptable", "Poor"))
df$pct_change <- df$pct_change - 100
df$`Percentage Change` <- df$pct_change

# Plot
ggplot(data = df, aes(x = Time, y = `Percentage Change`, group = Group, colour = Group)) +
    geom_line(aes(linetype = Group), size = 1) + geom_point() + scale_color_manual(values = c("green4",
    "orange1", "red3")) + facet_wrap(. ~ GV_Index, dir = "h")
```

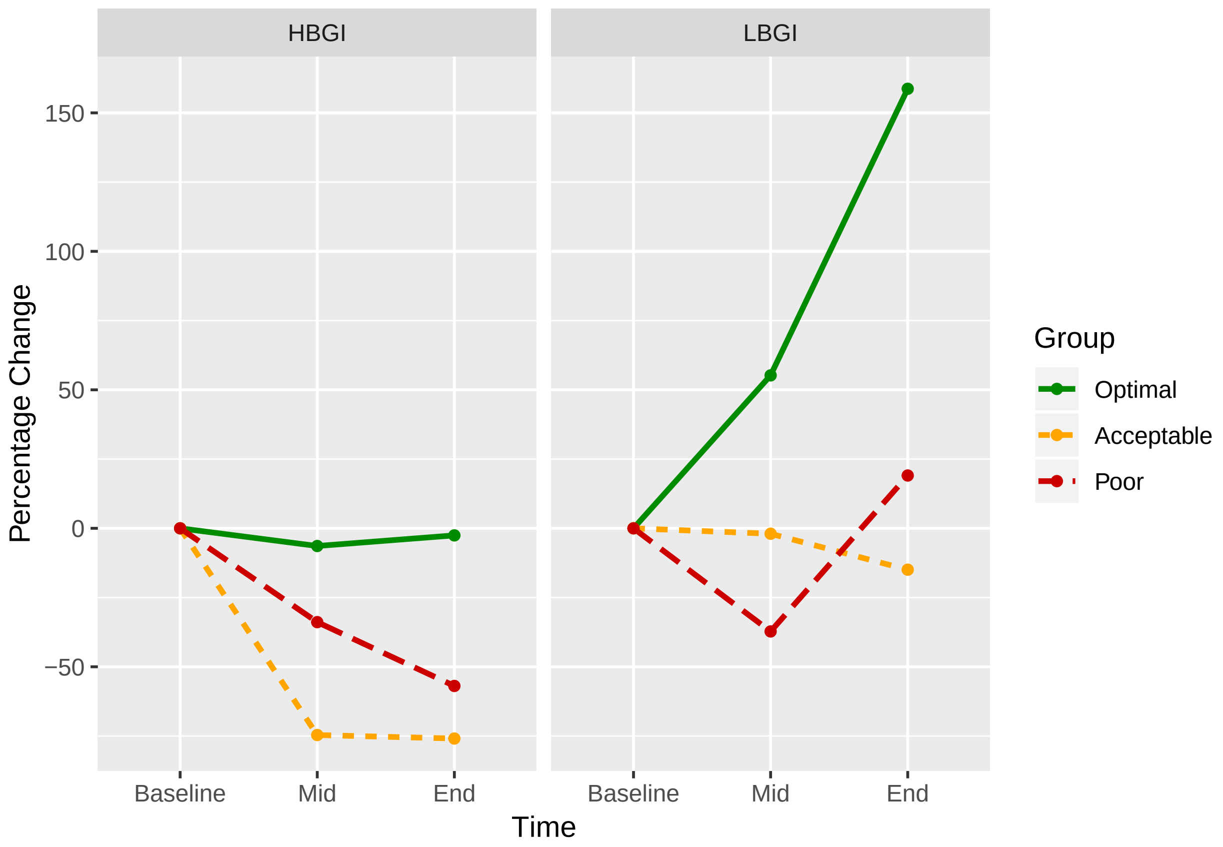

5.2 Pioncare Plot

The Poincare Plot has been achieved with the `gglagplot` function in `ggplot2` package using the `ggthemes` library. The data was cleaned for missing observations and were segregated by the time of intervention *(Start, Mid Intervention, End)*. The data was also categorized according to the HbA1c control *i.e.* Optimal Control Group, Acceptable Control Group and Poor Control Group. Each of the plot was then arranged in a grid using the `gridExtra` package.

```
# Load Dataset
df_lagplots <- read_csv("C:/Users/Arun/Dropbox/CGM_Study/data_analysis/Final_Data_with_Imputations.csv")
# The path to the data should be changed depending on its location on the
# computer

# Split data according to Baseline Group
df_lagplots_o <- df_lagplots[which(df_lagplots$Baseline_Group == "Optimal Control"),
    ]
df_lagplots_a <- df_lagplots[which(df_lagplots$Baseline_Group == "Acceptable Control"),
    ]
df_lagplots_p <- df_lagplots[which(df_lagplots$Baseline_Group == "Not Controlled"),
    ]

# Drop 10050 from poor group (incomplete/missing observations)
df_lagplots_p <- df_lagplots_p[df_lagplots_p$dc_number != 10050, ]

# Optimal Group
```

```r
df_lagplots_o_1 <- df_lagplots_o[which(df_lagplots_o$date_index == 2), ]
df_lagplots_o_2 <- df_lagplots_o[which(df_lagplots_o$date_index == 7), ]
df_lagplots_o_3a <- df_lagplots_o[which(df_lagplots_o$date_index == 13), ]
df_lagplots_o_3b <- df_lagplots_o[which(df_lagplots_o$dc_number == "10141" &
    df_lagplots_o$date_index == 10), ]
df_lagplots_o_3c <- df_lagplots_o[which(df_lagplots_o$dc_number == "10150" &
    df_lagplots_o$date_index == 12), ]
df_lagplots_o_3 <- rbind.data.frame(df_lagplots_o_3a, df_lagplots_o_3b, df_lagplots_o_3c)

# Acceptable Group
df_lagplots_a_1 <- df_lagplots_a[which(df_lagplots_a$date_index == 2), ]
df_lagplots_a_2 <- df_lagplots_a[which(df_lagplots_a$date_index == 7), ]
df_lagplots_a_3a <- df_lagplots_a[which(df_lagplots_a$date_index == 13), ]
df_lagplots_a_3b <- df_lagplots_a[which(df_lagplots_a$dc_number == "10118" &
    df_lagplots_a$date_index == 12), ]
df_lagplots_a_3 <- rbind.data.frame(df_lagplots_a_3a, df_lagplots_a_3b)

# Poor Group
df_lagplots_p_1 <- df_lagplots_p[which(df_lagplots_p$date_index == 2), ]
df_lagplots_p_2 <- df_lagplots_p[which(df_lagplots_p$date_index == 7), ]
df_lagplots_p_3 <- df_lagplots_p[which(df_lagplots_p$date_index == 13), ]

# Lagplot Optimal Group
df_o_1 <- (t(as.data.frame(split(df_lagplots_o_1$Glucose, 1:96))))
df_o_1_Glucose <- rowMeans(df_o_1)
names(df_o_1_Glucose) <- NULL

df_o_2 <- (t(as.data.frame(split(df_lagplots_o_2$Glucose, 1:96))))
df_o_2_Glucose <- rowMeans(df_o_2)
names(df_o_2_Glucose) <- NULL

df_o_3 <- (t(as.data.frame(split(df_lagplots_o_3$Glucose, 1:96))))
df_o_3_Glucose <- rowMeans(df_o_3)
names(df_o_3_Glucose) <- NULL

d_o <- cbind(df_o_1_Glucose, df_o_2_Glucose, df_o_3_Glucose)
d_o_new = d_o[seq(1, nrow(d_o), 5), ]
colnames(d_o_new) <- c("Start", "Mid", "End")

# Lagplot Acceptable Group
df_a_1 <- (t(as.data.frame(split(df_lagplots_a_1$Glucose, 1:96))))
df_a_1_Glucose <- rowMeans(df_a_1)
names(df_a_1_Glucose) <- NULL

df_a_2 <- (t(as.data.frame(split(df_lagplots_a_2$Glucose, 1:96))))
df_a_2_Glucose <- rowMeans(df_a_2)
names(df_a_2_Glucose) <- NULL

df_a_3 <- (t(as.data.frame(split(df_lagplots_a_3$Glucose, 1:96))))
df_a_3_Glucose <- rowMeans(df_a_3)
names(df_a_3_Glucose) <- NULL

d_a <- cbind(df_a_1_Glucose, df_a_2_Glucose, df_a_3_Glucose)
```

```r
d_a_new = d_a[seq(1, nrow(d_a), 5), ]
colnames(d_a_new) <- c("Start", "Mid", "End")

# Lagplot Poor Group
df_p_1 <- (t(as.data.frame(split(df_lagplots_p_1$Glucose, 1:96))))
df_p_1_Glucose <- rowMeans(df_p_1)
names(df_p_1_Glucose) <- NULL

df_p_2 <- (t(as.data.frame(split(df_lagplots_p_2$Glucose, 1:96))))
df_p_2_Glucose <- rowMeans(df_p_2)
names(df_p_2_Glucose) <- NULL

df_p_3 <- (t(as.data.frame(split(df_lagplots_p_3$Glucose, 1:96))))
df_p_3_Glucose <- rowMeans(df_p_3)
names(df_p_3_Glucose) <- NULL

d_p <- cbind(df_p_1_Glucose, df_p_2_Glucose, df_p_3_Glucose)
d_p_new = d_p[seq(1, nrow(d_p), 5), ]
colnames(d_p_new) <- c("Start", "Mid", "End")

# Final Plots
p1 <- gglagplot(d_o_new, lags = 1, xlim = c(50, 250), ylim = c(50, 250), colour = T,
    legend.position = "none")
p2 <- gglagplot(d_a_new, lags = 1, xlim = c(50, 250), ylim = c(50, 250), colour = T,
    legend.position = "none")
p3 <- gglagplot(d_p_new, lags = 1, xlim = c(50, 250), ylim = c(50, 250), colour = T,
    legend.position = "none")

grid.arrange(p1, p2, p3, ncol = 1, nrow = 3)
```

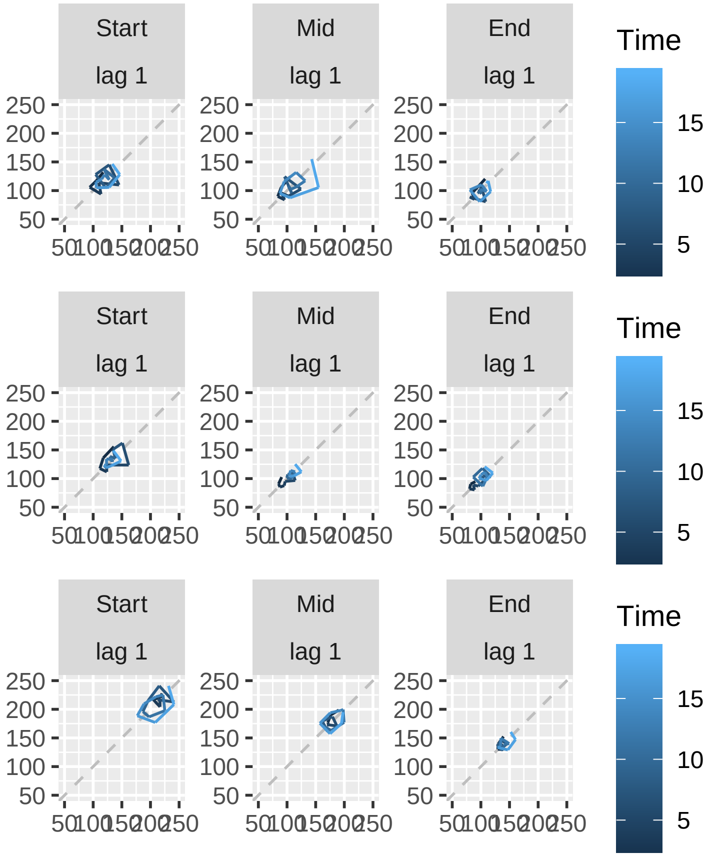
Start
lag 1
Mid
lag 1
End
lag 1
Time
15
10
5
250
200
150
100
50
50
100
150
200
250
